# Progress in Surgery

**Published:** 1897-09-25

**Authors:** 


### Progress in Surgery.

### ABDOMINAL SURGERY.

### Appendicitis.

The progress in the surgery of appendicitis is the outcome of bitter controversy, calumny, and undignified forms of argument. On the one side surgeons, disgusted by fatality from medical delay, urge early operation with dogmatic vehemence and unrestrained ardour, heedless of its inherent risks, its possible complications, and its sad results in unskilled hands. On the other side physicians, whose tender feelings are touched in the hour of dire distress, whose moral instincts are offended by precocious surgery (in but few cases though it be), whose consciences are smitten with obviously-misquoted statistics and misstated results, shrink from the only means of saving life. In the chaos of professional opinion, English and Continental, but particularly American, the foundations of the medical stronghold do shake, for dogmas are founded upon a disease which, chameleon-like, has many aspects, not the same to the family physician and the far-seeing pathologist.

What we want in appendicitis is more consensus of opinion medically and surgically, more constant observation in the early stages, and more considerate and congenial consultation between the ablest *confrères*.

A complete list of references will be found at the end of this *résumé*, which he who runs may read. The controversial points are not incorporated in the paper, but are placed separately in striking tabular contrast.

**Causes.**—These are given generally as, firstly, predisposing—anatomical and vestigial characters, dependent position, peculiar circulation, and traumatism; secondly, exciting—catarrhal, ulcerative, and infective forms. Traumatism and muscular action must be only minor causes, judging from the fact that at Rugby, Charterhouse, and John Watson's schools the affection has been comparatively rare. The internal traumatism of concretions and entozoa comes under a different heading.

The appendix is very vulnerable to infective processes. Its outer peritoneal coat does not expand, and when its lymphoid tissue swells it does so centripetally, blocking the lumen and causing compression anæmia of the walls. Moreover, its vascular supply is a single artery, and when infective thrombosis occurs, gangrene of the appendix must result, which then becomes a prey to the bacilli and a factory for the toxins.

Dieulafoy holds that appendicitis is always due to the transformation of the canal of the appendix into a closed cavity by some means or other, by fæcal matter, a concretion, a calculus, catarrh of the walls, a fibrous stricture, or a foreign body.

Robinson (Chicago) explains the infrequency of appendicitis in women by the fact that in many women the appendix hangs down in the pelvis, and is there protected from traumatism inflicted by the psoas muscle.[5 7 9 11 13 14]

### The Appendicitis Controversy.—Contrasted Opinions.

**Causes.**—Appendicitis is always the result of the transformation of the canal of the appendix into a closed cavity. This transformation may take place at any point in the canal. The symptoms of appendicitis do not declare themselves until the formation of a closed cavity is accomplished. This is the "accès appendiculaire."—*Prof. Dieulafoy.*

In appendicitis it is not necessary to have anything in the appendix except bacteria.—*Morris, New York.*

I have "found cases of perforation where the appendix was dilated in all its extent."—*Dr. Laveran.*

In only three cases did I find the appendix closed.—*Dr. Broca.*

I have operated in fifty-four cases, and in majority found the appendix perfectly permeable.—*Dr. Tuffier.*

It is not at all rare to have complete closure of the appendix without any inflammation.—*Dr. Poucet.*

**Pathology.**—Catarrhal appendicitis furnishes the greater number of cases of recurrent appendicitis, and this is the variety which may, and usually does, recover without surgical treatment.—*Mayo Robson.*

"Colic of the appendix" does not exist. This term perpetuates an error—it ought to disappear.—*Dieulafoy.*

Those of us who are in the habit of getting the specimens find that the cases diagnosticated at the bedside as catarrhal appendicitis are cases with big or little concretions, sloughs, &c.—*Morris, New York.*

There can be little doubt that this was a case of "appendicular colic"; the griping pains described by the patient and the muscular hypertrophy being due to the efforts of the distal portion to empty itself through the constriction caused by the kink.—*Hare, Australia.*

Reflex, and dependent upon appendicular colic.—*In Hysteria-Rendu, Talamon.*

**Diagnosis.**—For every case of "appendicitis" I have been called upon to treat I have met with at least five cases "with symptoms so closely resembling those of inflammation of the appendix that it was impossible to say that appendicitis did not exist until free purgation has been used, when there was ample proof that other causes, entirely separate from the appendix, were at the bottom of the sickness."

It is the general opinion that purgatives are not safe in cases of supposed appendicitis, enemata being usually recommended instead.—*Vide Text-books.*

**Mortality.**—I am able to say that of about one hundred cases not subjected to operation the direct mortality was less than 10 per cent. A considerable number made complete recoveries.—*Dr. Jas. Dunn, Minneapolis.*

Treves and other English surgeons have shown that 80 per cent. of patients with appendicitis got well without operation. One-third of the bodies in my dissecting-room had shown evidences of previous inflammatory trouble around the appendix, but had died of other diseases.—*Dr. F. W. McRae.*

I honestly believe that the mortality arising from operative surgery would be decreased if the physician were given a little more consideration.—*F. Halton, Brooklyn.*

The mortality, even where an early operation is performed, will be from 12 to 15 per cent. An operation is necessary in at least one-half of all cases.—*Dr. Seth Mereness.*

Of 213 cases in the wards, medical and surgical, the average mortality was 8·9 per cent. Nineteen ended fatally. Diffuse peritonitis was present in 21 cases, early operation was performed, and 14 were fatal; 82 per cent. of 192 cases of circumscribed peritonitis yielded to non-operative treatment. Of the remaining 18 per cent., 33 had operation with 2 deaths. Three died without operation, which if performed would probably have prevented death.—*J. Rotter, Berlin.*

Combined statistics of Rotter and Sonnenberg gave spontaneous cure in 84 per cent. of *all* cases.—*J. Rotter.*

The aggregate mortality at St. Bartholomew's and St. Thomas's Hospitals appears to be about 20 per cent.

My investigations lead me to believe that a fair estimate of the (ultimate) mortality from appendicitis treated medically is 25 per cent., which would give a total of 51,500 deaths annually in the United States. Among my own cases of appendicitis, treated surgically, the death-rate is only 2 per cent. The surgical deate-rate of 2 per cent. could evidently have been avoided if the two cases could have had operation at a time which I would have chosen.—*Morris, New York.*

It is true some cases will eventually recover by medical treatment (sixteen to four hundred according to Ribberts), and a slightly greater number will apparently recover from an attack.—*Deaver, "A Treatise on Appendicitis," 1896.*

I have had six cases that were reported in the *Buffalo Medical Journal*, February, 1896; two died, three made unsatisfactory recoveries. Only the operated case made a satisfactory recovery.—*Dr. Toms, Bellport.*

In walled-off abscesses medicine has a mortality of 75 per cent., against surgery's 20 per cent.—*Johnston.*

In spite of the advances in surgical treatment, the death-rate from appendicitis is on the increase.—*Dr. North, Brooklyn.*

**Necessity for Operation.**—The statistics of Toftt and the general experience of the profession prove that in a very large proportion of cases of appendicitis there never arises the consideration of the question of surgical interference. But these mild cases and their progress throw into strong relief the events in others. I have seen very many of such cases get thoroughly and permanently well.—*MacDougall Cannes.*

Too early intervention is not without danger, being liable to spread infection.—*Dr. Le Dentu.*

If there are many cases to be treated surgically there are as many to be treated by the physician.—*Dr. Le Dentu.*

In every undoubted case of appendicitis, however slight may be the symptoms, the appendix should be removed at once.—*Dr. Pozzi.*

I assert with full conviction that no medical treatment for appendicitis exists.—*Dieulafoy.*

I believe every case of appendicitis should be operated upon as soon as the diagnosis has been made, provided only that competent surgical aid can be obtained.—*Morris, New York.*

**Operative Sequelæ.**—In order to avoid abdominal scars and liability to ventral hernia.—*Morris, N.Y.*

Every surgeon of experience knows how unfortunate a patient is who has a ventral hernia following.—*Wright, Brooklyn.*

No harm is done by opening the peritoneal cavity. *Dr. Gollyer, New York.*

**Recovery after General Peritonitis.**—Usually the patients who recovered from general suppurative peritonitis, and whose surgeons believed they had saved them by operation, did not belong among the worst class. They were usually cases either of fibrinous peritonitis or of suppurative peritonitis, which did not involve the whole peritoneal cavity.—*Dr. Andrew Smith, New York.*

In operating where there is general infective peritonitis we must be prepared to lose many patients. I have been fortunate in saving five out of six cases of this kind on which I have operated during the year.—*Mayo Robson, B.M.J., December 19th, 1896.*

Note.—One of these cases is reported thus: "Abdomen *practically* filled with pus."

**The Cause of Pain.**—The pains in the first phase of appendicitis are the consequence of the transformation of the canal into a closed cavity, according to Dieulafoy, just as occurs in the cavity of the tympanum when it is transformed into a closed cavity by obstruction of the Eustachian tube.

In chronic appendicitis the cause of the pain in walking, &c., is probably due to the stretching of the adhesions by the psoas muscle, the cæcum and appendix lying in contact with its longest range of action. The removal of the bean-sized exudate about an appendix adherent to the psoas is said to have relieved a patient of sixteen months' suffering on walking.[8 11]

*(To be continued.)*

